# “Self” versus “Non-Self” Connectivity Dictates Properties of Synaptic Transmission and Plasticity

**DOI:** 10.1371/journal.pone.0062414

**Published:** 2013-04-29

**Authors:** Huisheng Liu, Edwin R. Chapman, Camin Dean

**Affiliations:** 1 Department of Neuroscience and Howard Hughes Medical Institute, University of Wisconsin, Madison, Wisconsin, United States of America; 2 The European Neuroscience Institute Göttingen (ENI-G), Göttingen, Germany; University of Nebraska Medical Center, United States of America

## Abstract

Autapses are connections between a neuron and itself. These connections are morphologically similar to “normal” synapses between two different neurons, and thus were long thought to have similar properties of synaptic transmission. However, this has not been directly tested. Here, using a micro-island culture assay in which we can define the number of interconnected cells, we directly compared synaptic transmission in excitatory autapses and in two-neuron micronetworks consisting of two excitatory neurons, in which a neuron is connected to one other neuron and to itself. We discovered that autaptic synapses are optimized for maximal transmission, and exhibited enhanced EPSC amplitude, charge, and RRP size compared to interneuronal synapses. However, autapses are deficient in several aspects of synaptic plasticity. Short-term potentiation only became apparent when a neuron was connected to another neuron. This acquisition of plasticity only required reciprocal innervation with one other neuron; micronetworks consisting of just two interconnected neurons exhibited enhanced short-term plasticity in terms of paired pulse ratio (PPR) and release probability (Pr), compared to autapses. Interestingly, when a neuron was connected to another neuron, not only interneuronal synapses, but also the autaptic synapses on itself exhibited a trend toward enhanced short-term plasticity in terms of PPR and Pr. Thus neurons can distinguish whether they are connected via “self” or “non-self” synapses and have the ability to adjust their plasticity parameters when connected to other neurons.

## Introduction

Neuronal circuits are generally thought of as collections of neurons connected to eachother by interneuronal synapses. But in addition to these connections neurons can also form autapses or “self synapses”: connections between a neuron and itself. Autapses are relatively common in many brain regions [1], [2], with some classes of neurons exhibiting extensive self-innervation [3]. For example, fast-spiking interneurons in the neocortex are self-innervated by GABAergic autaptic connections that regulate spike timing to promote temporal precision of synaptic transmission [4], [5]. Inhibitory autaptic synapses provide a self-stabilizing negative feedback influence on circuits, but excitatory autapses also exist. Excitatory autapses in certain Aplysia neurons, for instance, cause persistent activity essential for the initiation and maintenance of feeding behavior [6].

Autaptic synapses in the brain have been identified morphologically by injecting neurons with intracellular markers. These synapses appear morphologically similar to interneuronal synapses [1] and thus have been assumed to have similar basic properties compared to interneuronal synapses. In fact, autapses, single neurons grown on small islands of substrate in culture that form synapses only on themselves, have been widely used to study synaptic transmission, owing to the ease with which experimenters can both stimulate and record from the same cell, and thus assess presynaptic transmission mechanisms [7].

However, recent evidence suggests that autapses may have different properties from neurons that are assembled into networks. For example, in synaptotagmin-1 knockout neurons, where the fast calcium-sensor of neurotransmitter release is absent, different phenotypes are observed depending on whether recordings are made from autapses or from pairs of interconnected neurons [8], [9], [10], [11]. This suggests the hypothesis that fundamental properties of synaptic transmission may differ depending on whether “self” or “non-self” synapses are formed. However, this idea has not yet been tested. In the studies above, a whole-cell intercellular voltage change was used to stimulate syt-I knockout versus wild-type autapses, while an extracellular voltage change was used to stimulate mass cultures [12]. Thus wild-type autaptic and mass cultures were not directly comparable.

Here, we used whole cell recordings from single neurons on islands, or double whole cell patch clamp from each of two interconnected neurons growing on two-neuron islands, to directly compare autaptic and interneuronal connections using identical stimulation paradigms. We investigated basic properties of synaptic transmission and synaptic plasticity in this reduced micro-island hippocampal neuron culture system in which we compared autapses (single neurons innervating only themselves), to both autaptic and interneuronal synapses in “networks” of just two neurons.

Interestingly, we found that autaptic synapses exhibited enhanced EPSC amplitude, charge, and RRP size compared to interneuronal synapses. But autaptic synapses exhibited deficiencies in plasticity in comparison to interneuronal synapses. Micronetworks consisting of two interconnected neurons exhibited enhanced short-term plasticity in terms of paired pulse ratio and release probability, compared to autapses. Thus “self” versus “non-self” synapses exhibit fundamental differences in synaptic transmission and plasticity.

## Materials and Methods

### Ethics Statement

All research involving animals was done in accordance with the guidelines of the National Institutes of Health, as approved by the Animal Care and Use Committee of the University of Wisconsin, Madison.

### Hippocampal Neuron Micronetwork Cultures

For autaptic cultures, and two-neuron micronetworks, hippocampal neuron cultures were prepared as described previously [7], [13]. Briefly, 12 mm coverslips in 24-well culture plates were coated with 0.15% agarose. Microdrops of 0.25 mg/ml collagen and 0.5 mg/ml poly-lysine were then sprayed on top of the agarose using a microatomizer to generate “islands” of substrate varying in size from 100–1000 µm in diameter. Hippocampi of E18 Wistar rats were isolated following CO_2_ euthanasia of pregnant rats and decapitation of embryos, as described previously [14], in accordance with the guidelines of the National Institutes of Health, as approved by the Animal Care and Use Committee of the University of Wisconsin, Madison. Dissociated hippocampal neurons were resuspended at a concentration of 5–8×10^4^/ml and 0.5 ml was plated per well. This resulted in several islands containing one or two neurons. One and two-neuron micronetworks were identified by light microscopy prior to electrophysiological experiments. For dissociated cultures used in FM dye experiments, neurons were plated at a density of 80,000 cells/cm^2^ on coverslips coated with 0.5 mg/ml poly-lysine, washed once with water and dried briefly, prior to plating cells. In all cases, cells were grown in Neurobasal supplemented with 2% B-27 and 2 mM Glutamax (Gibco/Invitrogen).

### Electrophysiology

**Figure 1:**
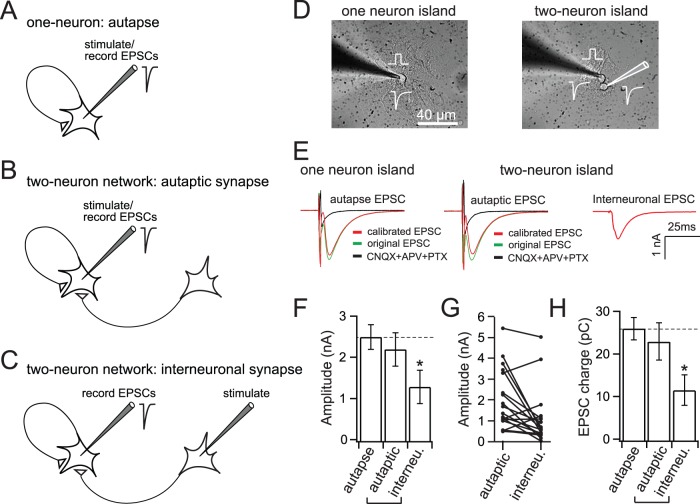
Autaptic synapses have larger EPSCs than interneuronal synapses. (A) Cartoon of an autapse, in which a single neuron growing on a micro-glial island innervates itself, and thus can be stimulated and recorded from at the same time. (B) Recording scheme for autaptic synapses, and interneuronal synapses (C) in two-neuron micronetworks. (D) Image of micro-island cultures consisting of one (left panel) or two (right panel) hippocampal neurons. (E) Representative single EPSC recordings from an autapse (left) and from autaptic or interneuronal synapses in two-neuron micronetworks. The autaptic response (calibrated EPSC) was calculated by subtracting the offset current, due to the action potential preceding the EPSC, from the original EPSC; the offset current was determined by adding CNQX to block AMPA receptors. (F) EPSC amplitude in single neuron autapses (2.5±0.3 nA), and in autaptic (2.2±0.4 nA), and interneuronal (1.3±0.4 nA) synapses in two-neuron micronetworks. Autaptic synapse EPSC amplitude in both one and two-neuron micro-islands is larger than interneuronal EPSC amplitude. (G) Pairwise plot showing autaptic and interneuronal amplitudes recorded from neurons in two-neuron networks: 75% of pairs exhibited higher EPSCs in autaptic synapses than in interneuronal synapses, where interneuronal synapses had 66.9±13.1% of the EPSC amplitude of autaptic synapses. (H) EPSC charge in autapses (26.0±2.6 pC) and autaptic synapses in two-neuron networks (23.0±4.4 pC), is significantly larger than in interneuronal synapses (11.7±3.6 pC). Statistical significance was determined by one-way ANOVA and Newman-Keuls post-hoc test. * = p<0.05, n = 18 (autapses), 20 (autaptic synapses in two-neuron micronetworks), 20 (interneuronal synapses in two-neuron networks). All data shown represent mean ± SEM.

Double whole-cell patch-clamp recordings were made from micro-island cultures in which only one or two excitatory neurons were harbored per island. Islands containing inhibitory neurons, which exhibited no response in the presence of picrotoxin, were not analyzed. Autapse EPSCs were recorded from single neurons on islands, and both autaptic and interneuronal EPSCs were recorded from each of the two interconnected neurons for two-neuron islands. The autaptic response was calculated after subtracting the offset current due to the action potential (seen as the spike preceding the EPSC in Figure 1E autapse and autaptic examples). The offset current due to the action potential was determined by adding CNQX to block AMPA receptors following EPSC recordings. Spontaneous mEPSCs were recorded prior to stimulation, since autapses and two-cell micronetworks did not have spontaneous action potentials. All recordings were performed 12–15 days after neurons were plated on coverslips. The pipette intracellular solution consisted of 130 mM K-gluconate, 1 mM EGTA, 5 mM Na-phosphocreatine, 2 mM Mg-ATP, 0.3 mM Na-GTP, and 10 mM HEPES, pH 7.3 (290 mOsm). The extracellular solution consisted of 140 mM NaCl, 5 mM KCl, 2 mM CaCl_2_, 1 mM MgCl_2_, 10 mM glucose, 10 mM HEPES, pH 7.3 (300 mOsm), 50 µM D-2-amino-5-phosphonopentanoate (D-AP5), and 0.1 mM picrotoxin. Picrotoxin was dissolved in ethanol, and D-AP5 was dissolved in NaOH. All drugs were from Sigma-Aldrich. Neurons were voltage clamped at −70 mV with an EPC-10/2 amplifier (HEKA). Only cells with series resistances of <15 MΩ, with 70–80% of this resistance compensated, were analyzed. Currents were acquired using PATCHMASTER software (HEKA), filtered at 2.9 kHz, and digitized at 10 kHz. Data were analyzed using Clampfit (Molecular Devices), and Igor (Wavemetrics). All experiments were performed at room temperature.

### Counting the Number of Synapses in Micronetworks

12–14 DIV neurons growing on coverslips were fixed with 4% paraformaldehyde in PBS, permeabilized and blocked in 0.1% Triton X-100 and 10% goat serum, and immunostained with guinea pig anti-VGluT1 (Millipore) and Cy2 anti-guinea pig secondary antibody (Jackson ImmunoResearch Laboratories) to mark excitatory presynaptic terminals. Coverslips were mounted in Fluoromount (Southern Biotechnology Associates) and images were acquired with identical laser power and gain settings on an Olympus FV1000 upright confocal microscope with a 60X 1.10 NA water-immersion objective. The number of synapses was calculated using MetaMorph software, with thresholds such that all recognizable synaptic puncta were selected.

### FM1-43 Dye Loading Experiments

Boutons in autaptic or dissociated hippocampal cultures were loaded with 10 µM FM1-43 (Invitrogen) in 45 mM KCl depolarizing buffer (100 mM NaCl, 45 mM KCl, 2 mM CaCl_2_, 2 mM MgCl_2_, 5.5 mM glucose, 20 mM HEPES, pH 7.3) for 2 min. Coverslips were then washed with 5 mM low K^+^ buffer (140 mM NaCl, 5 mM KCl, 2 mM CaCl_2_, 2 mM MgCl_2_, 5.5 mM glucose, 20 mM HEPES, pH 7.3) for 5 min to remove excess dye. Coverslips were placed in live imaging chambers (Warner Instruments) and FM1-43 fluorescence images were acquired with a Cascade II EMCCD camera (Roper Scientific Photometric) on a Nikon TE300 inverted microscope. Images were collected with 200 ms exposures at 1 s intervals during dye destaining. A baseline of 10 images was collected before addition of depolarizing buffer to destain boutons. Dye-labeled boutons were selected as regions of interest in MetaMorph (Improvision), and fluorescence intensity plotted versus time. Recycling vesicle pool size was determined by the total fluorescence loss from boutons during destaining (final fluorescence following destaining subtracted from starting fluorescence before addition of 45 mM KCl).

## Results

To test the idea that synaptic properties may differ in “self” versus “non-self” synapses we employed a micronetwork culture system using hippocampal neurons, where single cell autapses or two interconnected neurons were grown on micro-islands. After 12–15 days *in vitro*, we examined the basic properties of synaptic function using whole-cell patch clamp recordings from autapses, and double whole-cell patch clamp paired recordings from two-neuron networks on islands. We compared autapses, in which a single neuron is connected only to itself (Fig. 1A), to two-neuron networks in which each neuron can form synapses on itself (autaptic synapses, Fig. 1B) or on the other neuron (interneuronal synapses, Fig. 1C) in islands of two interconnected neurons (Fig. 1D). We first compared responses to a single action potential (AP) (Fig. 1E). We found that the amplitude of excitatory post-synaptic currents (EPSCs) of autaptic synapses in both single neurons and in two-neuron micronetworks, were significantly larger than those of interneuronal synapses formed between two interconnected neurons (Fig. 1F). This is similar to a previous report in which evoked autaptic currents were enhanced compared to currents recorded from mass cultures [15]. In 75% of pairs (15 out of 20) autaptic synapses exhibited higher EPSCs than interneuronal synapses (Fig. 1G). Interneuronal synapses had 66.9±13.1% of the EPSC amplitude of autaptic synapses. The EPSC charge of autaptic synapses, in both single neuron and two-neuron islands, was also significantly larger than that of interneuronal synapses (Fig. 1H).

**Figure 2:**
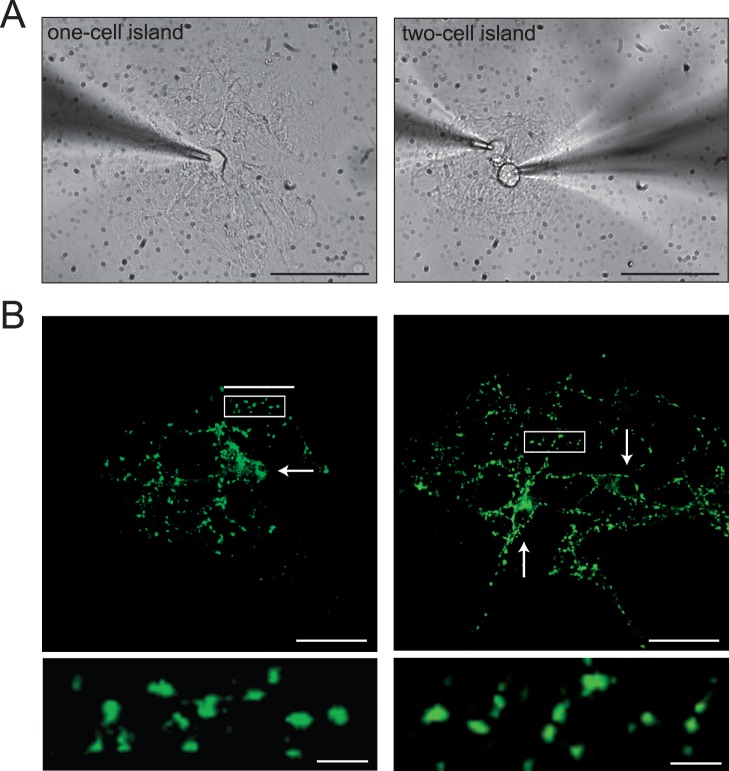
The number of synapses per neuron is constant in one and two-neuron micronetworks. (A) Bright-field images of one- and two-neuron micronetworks with patch clamp recording electrodes in place. Scale bars are 40 µm. (B) VGluT1 immunostaining of glutamatergic synapses in microislands containing one or two neurons (white arrows). The lower panels are enlarged images of the indicated rectangular areas D, The number of synapses in one- and two-neuron microislands is indicated: 330±70 synapses in one-neuron islands (n = 9), and 750±120 synapses in two-neuron islands (n = 7). Scale bars are 40 µm in upper panels and 5 µm in lower panels.

This increase in EPSC amplitude could be due to an increase in the number of release sites (N), increase in size of quantal response (q) or increase in release probability (p) [16]. We previously found that the total number of synapses, N, in micro-island cultures is constant [8]. Here we repeated these experiments and verified that two-neuron networks had approximately twice the total number of synapses of single neuron autapses: 330±70 synapses in single neuron islands and 750±120 synapses in two-neuron islands (Fig. 2). Thus, if all synapses have the same transmission properties, we would expect the autapse EPSC amplitude recorded from one-neuron islands to equal the sum of the autaptic and interneuronal amplitudes recorded from neurons in two-neuron islands. But this is not the case. The autapse EPSC amplitude is approximately 2.5 nA and the sum of the autaptic (2.2 nA) and interneuronal (1.3 nA) amplitude is 3.5 nA. Likewise, the autapse EPSC charge is 25 pC and the sum of the autaptic (23 pC) and interneuronal (11 pC) synapse charge is 34 pC. Thus, the increase in EPSC size for autaptic synapses cannot be due to alterations in N.

**Figure 3:**
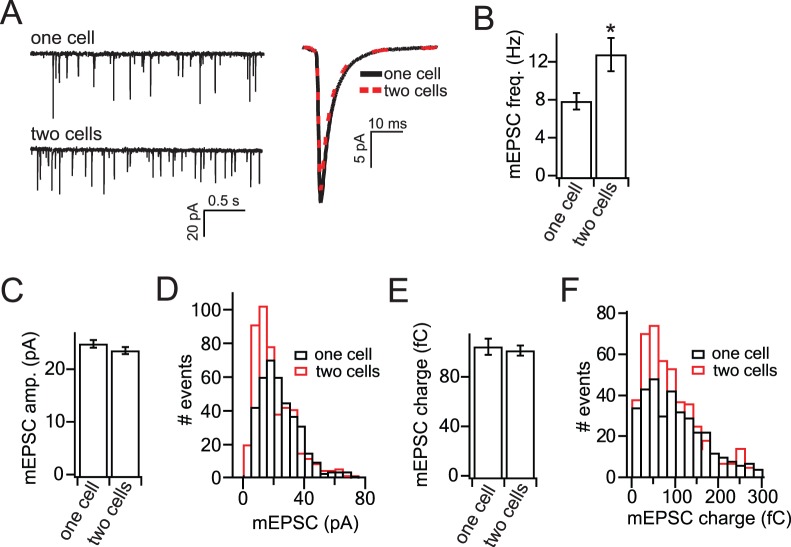
Autapses have reduced mEPSC frequency but no change in mEPSC amplitude, compared to neurons in two-cell micronetworks. (A) Representative mEPSC recordings from neurons in one-cell or two-cell micronetworks. (B) Average mEPSC frequency recorded from one-cell island autapses and from neurons in two-cell micronetworks. (C) Average mEPSC amplitude in one-cell autapses and from neurons in two-cell micronetworks. (D) Distribution of mEPSC amplitudes in one and two-cell networks (bin = 5 pA). (E) Average mEPSC charge recorded from one-cell and two-cell micronetworks. (F) Distribution of mEPSC charge in one and two-cell networks (bin = 20 fC). For B, C, and E, n = 18 autapses and 20 neurons from two-neuron micronetworks. For distributions in D and F, n = 400 mEPSCs from one-cell islands and 470 mEPSCs from two-cell islands. Statistical significance was determined by Student’s t-test (B, C, E) or Kolmogorov-Smirnov test (D, F). * = p<0.05. All data shown represent mean ± SEM.

To test if the increased EPSC size in autapses is due to an increase in q, the quantal response, corresponding to the number of surface-expressed post-synaptic receptors, we compared mEPSCs from neurons in one and two-neuron networks (Fig. 3A). Neurons in two-neuron islands had a higher mEPSC frequency compared to single neuron islands (Fig. 3B), as previously reported [8]. However, there was no significant difference in mEPSC amplitude between autapses and two-neuron micronetworks (Fig. 3C). Although we cannot distinguish between self and non-self inputs in the case of recording from a neuron in a two-neuron micronetwork, we assume that both types of synapses contribute to the average mEPSC amplitude. If different numbers of receptors are present at autaptic versus interneuronal synapses, this will generate two different populations of mEPSC amplitudes recorded from neurons in two-neuron networks. To determine if this is the case, we plotted distributions of mEPSC amplitudes recorded from neurons in one and two-neuron islands, but found only a single population of amplitudes in each case (Fig. 3D) and no evidence for autaptic versus interneuronal mEPSC amplitude being different. This was also true for mEPSC charge, which was the same for mEPSCs recorded from one and two-cell islands (Fig. 3E) with no distinct populations detected (Fig. 3F). Thus, the increased EPSC size recorded from autapses and autaptic synapses compared to interneuronal synapses is not due to an increase in post-synaptic receptors.

**Figure 4:**
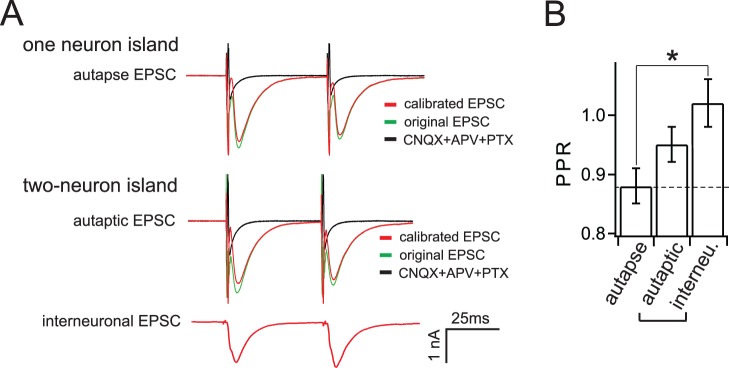
Interneuronal synapses display an enhanced paired-pulse ratio (PPR) compared to autapses. (A) Representative paired-pulse EPSCs recorded from autapses and autaptic and interneuronal synapses in two-neuron micronetworks. The calibrated EPSC was calculated by subtracting the offset current, determined by stimulation in the presence of CNQX to block AMPA receptors, from the original EPSC. (B) The PPR, determined by dividing the second EPSC by the first, is larger in two-neuron micronetworks (autaptic: 0.95±0.03; interneuronal: 1.02±0.04) than in autapses (0.88±0.03). Interneuronal synapses exhibited the highest PPR (n = 18 autapses, 20 autaptic synapses, and 20 interneuronal synapses. Statistical significance was determined by one-way ANOVA and Newman-Keuls post-hoc test. *p<0.05. All data shown represent mean ± SEM.

We next tested the paired pulse ratio (PPR), a measure of presynaptic plasticity, in autapses and in autaptic and interneuronal synapses in two-neuron micronetworks. The PPR is determined by the ratio of the amplitude of the second response to that of the first, following stimulation of a synapse twice in rapid succession, with a 50 msec interval [17]. Synapses with a high release probability (p) are likely to release more vesicles in response to the first stimulus, and have less available for release in response to the second, and therefore have a lower PPR. Conversely, low release probability corresponds to a high PPR and a higher degree of facilitation [17], [18]. The PPR of interneuronal synapses in two-neuron networks was significantly higher than that of autapses (Fig. 4A, B), consistent with earlier reports [19], indicating that autaptic synapses have a higher release probability than interneuronal synapses. Interestingly, autaptic synapses in two-neuron networks also exhibited an increase in PPR in comparison to autaptic synapses in single neurons, although this increase did not reach significance (p = 0.064) (Fig. 4A, B).

**Figure 5:**
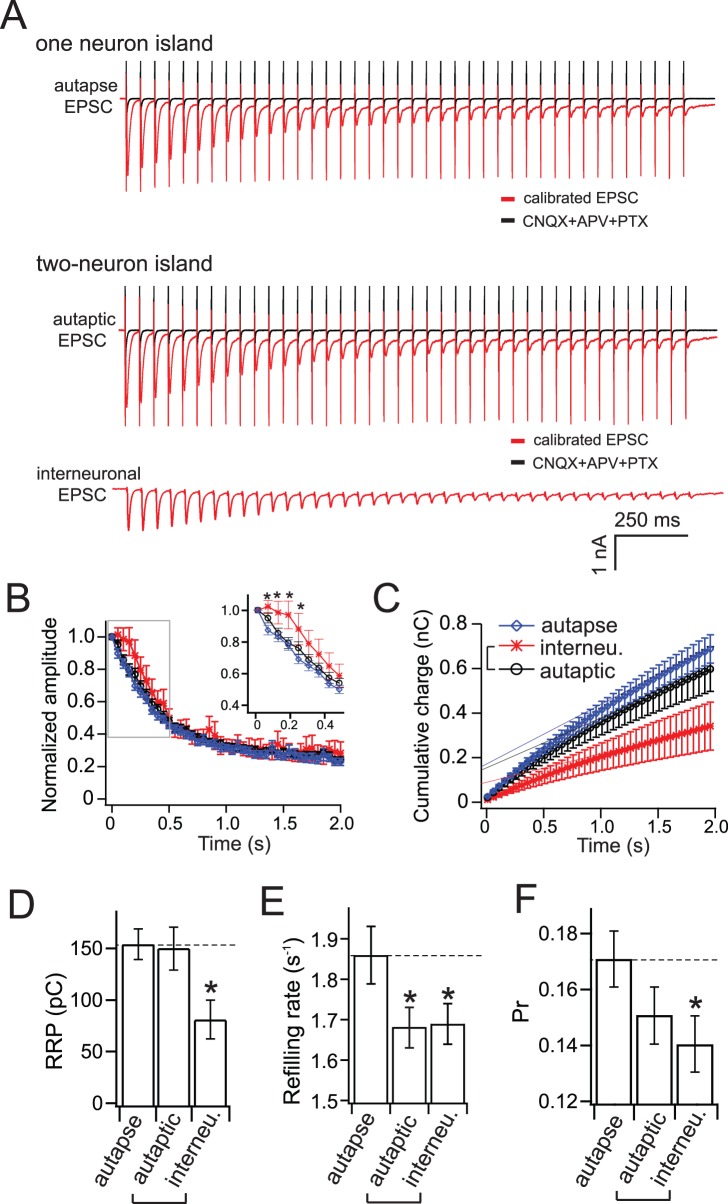
Synapses in two-neuron micronetworks display increased short-term plasticity, compared to autapses. (A) Representative EPSCs triggered by high-frequency stimulation (HFS, 40 APs/2 s) of autapses and two-neuron micronetworks. Calibrated EPSCs were calculated by subtracting the offset current, determined in the presence of CNQX to block AMPA receptors, from the original EPSC. (B) The peak amplitude of each EPSC normalized to the peak of the first response and plotted versus time. Note that autapses and autaptic synapses underwent immediate depression after the first few APs, compared to interneuronal synapses. Inset is a magnification of the graph in the indicated box, showing the responses to the first 10 stimuli. (C) Plot of cumulative total charge versus time. Data points from the 30^th^ to the 40^th^ EPSC were fitted with a linear function. The y-intercept is a measure of the RRP size (D), and the slope divided by the RRP size (to eliminate any influence of different RRP sizes between groups) reveals the SV refilling rate (E). (D) The RRP size in interneuronal synapses is smaller (81.8±18.7 pC) than in autaptic synapses in two-neuron networks (150.3±20.5 pC) or in autapses (153.9±14.7 pC). (E) The synaptic vesicle refilling rates during HFS were significantly reduced in both interneuronal and autaptic synapses of two-neuron micronetworks (autaptic: 1.68±0.05 s^−1^; interneuronal: 1.69±0.05 s^−1^) compared to autapses (1.86±0.07 s^−1^). (F) Release probability (Pr) was determined by dividing the single EPSC charge by RRP size. Two-neuron micronetworks exhibited reduced release probability in both autaptic (0.15±0.01) and interneuronal synapses (0.14±0.01) compared to autapses (0.17±0.01); the difference between autapses and autaptic synapses was not significant (p = 0.063). Statistical significance was determined by one-way ANOVA and Newman-Keuls post-hoc test. *p<0.05, n = 18 (autapses), 20 (autaptic synapses), and 20 (interneuronal synapses). All data shown represent mean ± SEM.

Only a small number of vesicles in the readily releasable pool (RRP) undergo exocytosis in response to an AP [20], [21]. The size of the RRP is a measure of synaptic strength and plasticity; synapses with more vesicles in the RRP have higher release probabilities [22]. In addition, the rate at which the RRP refills following depletion determines how quickly synapses depress during bursts of APs; if the RRP empties more quickly than it can be refilled, short-term depression results [17], [20], [23], [24]. Using high frequency train stimulation (40 APs/2s), we compared RRP size, refilling rate and release probability in autapses, and autaptic and interneuronal synapses in two-neuron networks (Fig. 5A).

Autapses and autaptic synapses underwent significantly greater depression in response to the first few APs during stimulation at 20 Hz, compared to interneuronal synapses (Fig. 5B). To determine RRP size, we fit the cumulative total charge with a linear function fitting to the last 10 stimuli (30^th^–40^th^) (Fig. 5C), where the y-intercept is a measure of the RRP size [25]. Interneuronal synapses exhibited a reduced RRP compared to autapses or autaptic synapses in two-neuron micronetworks (Fig. 5D). Interestingly, the synaptic vesicle refilling rate [26] (determined by the slope of the linear fit of cumulative charge in response to train stimulation divided by the RRP size) was reduced at both autaptic and interneuronal synapses in two-neuron networks, compared to autapses (Fig. 5E). Release probability, determined by single EPSC charge divided by RRP size [8], [27], was also significantly reduced in interneuronal synapses in two-neuron micronetworks compared to autapses (Fig. 5F). Release probability in autaptic synapses was intermediate, and reduced compared to autapses, although this reduction was not significant (p = 0.063).

**Figure 6:**
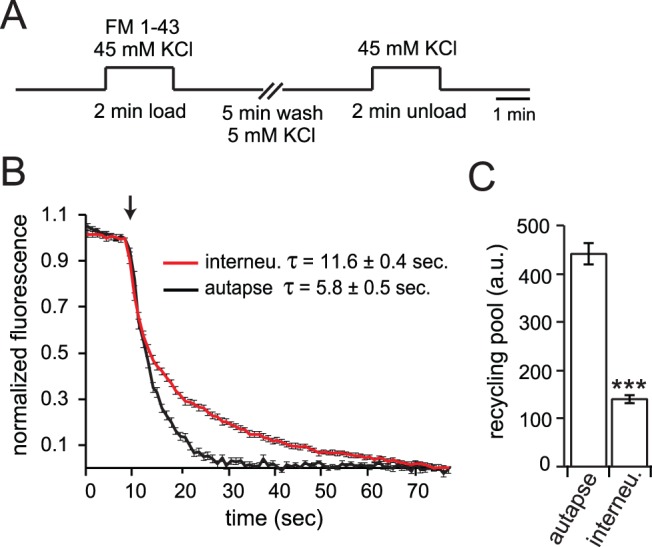
Analysis of synaptic vesicle recycling pool size and kinetics of exocytosis using FM dye. (A) Schematic of the FM1-43 dye loading and unloading protocol used to determine recycling vesicle pool size and destaining kinetics. Synaptic vesicles were loaded with FM1-43 for 2 min using 45 mM KCl, washed for 5 min and then unloaded with 45 mM KCl during image acquisition at 1 second intervals. (B), Quantitation of FM1-43 destaining in autaptic synapses and interneuronal synapses. A baseline of 10 images was collected prior to destaining by addition of 45 mM KCl, indicated by the arrow. (C) Recycling vesicle pool size calculated from the total FM1-43 fluorescence loss from boutons following stimulation for autaptic and interneuronal synapses. (n = 41; 5 different coverslips for autaptic synapses and n = 55; 6 different coverslips for interneuronal synapses). Statistical significance was determined by a Student’s t-test. ***p<0.001. All data shown represent mean ± SEM.

The RRP size and release probability can also be estimated by whole-cell recordings during the addition of sucrose, to specifically release only RRP vesicles [20], or by the use of FM dyes [28]. These methods cannot be used to distinguish autaptic and interneuronal synapses in two-neuron networks, since sucrose would affect both types of synapses and we cannot distinguish “self “from “non-self” FM dye loaded boutons. However, we analyzed recycling pool size in autapses compared to mass cultures, which presumably consist of predominantly interneuronal synapses (Fig. 6A). We found that autaptic synapses have a faster rate of FM dye destaining in response to stimulation, and a larger total recycling pool of vesicles compared to interneuronal synapses (Fig. 6B, C). This increase in FM dye destaining rate corresponds to a higher release probability, which is consistent with the measurement of RRP and release probability calculated from train stimulation.

## Discussion

Neuronal circuits contain both interneuronal and autaptic inputs. Here, we tested whether connectivity affects the properties of autapses. We found that autaptic synapses had enhanced EPSC amplitude, charge, and RRP size compared to interneuronal synapses, in both one and two-neuron micronetworks. Surprisingly, facilitation following repeated stimulation was absent in single neuron autapses and only became apparent when a neuron was connected to another neuron. Remarkably, this acquisition of plasticity only required reciprocal innervation with one other neuron; micronetworks consisting of just two interconnected neurons exhibited enhanced short-term plasticity in terms of PPR and release probability, compared to autapses.

Interestingly, once a neuron “talked” to another neuron, the properties of all of its synapses, even those formed on itself changed: the PPR and Pr of autaptic synapses in two-neuron networks showed an intermediate value between that of single-neuron autapses and interneuronal synapses, and the vesicle refilling rate, which limits the amount of transmitter released during long high frequency stimulation [20], was significantly reduced in both autaptic and interneuronal synapses in two-neuron networks, compared to single-neuron autapses.

The total number of synapses per neuron was constant in micro-networks (Figure 2) [8], and the quantal size (q) [16] was not significantly different between one and two-neuron islands (Figure 3), suggesting that the enhanced EPSC amplitude in autaptic synapses was not due to an increase in the number of synapses (N), or post-synaptic receptors (q). This leaves p, the release probability, as the dominant factor affecting EPSC size. Indeed, the release probability was increased in autapses compared to interneuronal synapses (Figure 5). This result was corroborated by FM dye destaining experiments, in which autapses had an increased recycling vesicle pool size (Figure 6) and rate of destaining compared to interneuronal synapes [8], corresponding to an increase in release probability [28], [29]. The release probability in turn affects synaptic plasticity at each synapse type: interneuronal synapses have a lower release probability and therefore a higher PPR than autaptic synapses.

Our results are consistent with previous reports in which evoked autaptic currents are enhanced compared to currents recorded from mass cultures [15], and the PPR of interneuronal synapses in two-neuron networks was significantly higher than that of autapses [19]. Our experiments extend these findings by comparing autapses in single neurons to autapses in two-neuron networks. This novel comparison revealed that when neurons are connected to other neurons, the properties of their autaptic synapses change.

The mechanisms that make “self” and “non-self” synapses different are unknown, but several possibilities exist. Presynaptic strength depends on the specific dendritic branch [29], with stronger terminals closer to the soma [30]. An axon emanating from a neuron may be more likely to encounter “self” dendrites than “non-self” dendrites, simply because of proximity, and therefore form autaptic synapses closer to the cell soma with higher release probability.An additional explanation could involve the maturation of synapses in the two different situations. Immature synapses tend to have a high release probability that becomes lower as synapses mature [31], [32]. Autapses may represent an “immature” synaptic state with a higher release probability than interneuronal synapses.

Classically, mEPSC rate and evoked release probability are expected to be correlated. However, interneuronal synapses have a higher mEPSC rate and lower evoked release probability than autaptic synapses. This might be explained by the recent discoveries that minis themselves regulate protein synthesis [33] and increased mini frequency correlates with decreased depression in response to evoked stimulation [34], [35]. In this case, an increase in mini rate in two-neuron networks would cause a decrease in evoked release probability, which is indeed what we observe. In addition, miniature and evoked responses have recently been reported in a number of studies to be mediated by distinct pools of vesicles [36], [37], [38], [39]. It is possible that these distinct vesicle pools are differentially regulated in autapses versus interneuronal networks.

Our data point to the post-synaptic site as the determinant of presynaptic release probability and vesicle-refilling rate: these parameters are altered depending on whether a synapse is formed on a neuron’s own dendrite or on that of another neuron. This suggests that a retrograde signal may be required, such as a BDNF, which can be released from post-synaptic sites (Lessmann) and affect presynaptic release probability [40].

The micro-network system we employed has the advantage of providing a direct comparison between autaptic and interneuronal synapses in a reduced system composed of a single excitatory neuron, or an excitatory neuron connected to one other excitatory neuron. However, in vivo networks of neurons are obviously much more complex and include multiple connections with a variety of cell types, including both excitatory and inhibitory neurons. It will be important in the future to test whether the same principles discovered here also apply to more complex networks in intact brain circuits. As an initial step, it would be interesting to test whether the same phenomenon occurs in GABAergic one and two-neuron micronetworks, or in micronetworks where one neuron is glutamatergic and one is GABAergic.

The micronetwork system could be further exploited to gain additional mechanistic insights. For example, autaptic versus interneuronal synapses could be visualized by expression of tagged pre and post-synaptic proteins [41] in one neuron of a two-neuron network, in conjunction with a marker of all synapses. One could then test whether autaptic synapses form preferentially at proximal or distal sites, which could determine their respective transmission properties. These experiments must be carefully controlled, however, to ensure the marker proteins do not perturb neuronal function. In addition, the effects of long-term activity blockade or increase during development could be tested in micro-networks, to determine the role activity plays in shaping autaptic versus interneuronal synapses in one and two-neuron networks.

Together our results imply that neurons have the ability to distinguish between “self” and “non-self” synapses and to adjust their plasticity parameters when connected to other neurons. It further suggests that interneuronal synapses are optimized to be plastic, while autaptic synapses are optimized for maximal reliability. Thus autaptic synapses may act to refine incoming signals from other neurons. Indeed, *in vivo* studies in neocortical fast-spiking interneurons suggests that autaptic connections, which are activated after each spike, are important for the precision of firing; blockade of these autaptic connections degrades temporal precision, and thus may compromise correct sensory representation [4], [5]. In addition, excitatory autaptic connections can lead to reliable persistent activity, which is important for functions as diverse as working memory [42] and the maintenance of feeding behavior [6], [43].

Interestingly, once a neuron “talks” to another neuron, all of its synapses, even those formed on itself became more plastic. Thus, neurons are not only able to distinguish “self” and “non-self” innervation, but are also able to distinguish when they are connected to other neurons and to adjust their transmission properties accordingly. These underlying principles may act to fine-tune the function of neuronal circuits.
